# Male involvement in family planning decision making in sub-Saharan Africa- what the evidence suggests

**DOI:** 10.11604/pamj.2014.19.349.5090

**Published:** 2014-12-03

**Authors:** Marius Zambou Vouking, Christine Danielle Evina, Carine Nouboudem Tadenfok

**Affiliations:** 1 Centre for the Development of Best Practices in Health, Yaounde Central Hospital, Henri-Dunant Avenue, Messa, Yaounde, Cameroon; 2 Central Regional Delegation, Ministry of Public Health, Yaounde, Cameroon; 3 Catholic University for Central Africa School of Health Sciences, Yaounde, Cameroon

**Keywords:** Male involvement, family planning decision making, Sub-Saharan Africa

## Abstract

The World Health Organization (WHO) estimated in 2012 that 287,000 maternal deaths occurred in 2010; sub-Saharan Africa (56%) and Southern Asia (29%) accounted for the global burden of maternal deaths. Men are also recognized to be responsible for the large proportion of ill reproductive health suffered by their female partners. Male involvement helps not only in accepting a contraceptive but also in its effective use and continuation. The objectives were to assess men's knowledge, attitude, and practice of modern contraceptive methods; determine the level of spousal communication about family planning decision making; and investigate the correlates of men's opinion about their roles in family planning decision making. We searched the following electronic databases from January 1995 to December 2013: Medline, Embase, CINAHL, LILAS, International Bibliography of Social Sciences, Social Services Abstracts, and Sociological Abstracts. Along with MeSH terms and relevant keywords, we used the Cochrane Highly Sensitive Search Strategy for identifying reports of articles in PubMed. There were no restrictions to language or publication status. Of 137 hits, 7 papers met the inclusion criteria. The concept of family planning was well known to men. In the Nigerian study, almost (99%) men were aware of the existence of modern contraceptives, and most of them were aware of at least two modern methods. Awareness of the condom was highest (98%). In the Malawi study, all of the participants reported that they were not using contraception before the intervention. In Ethiopia, above 90% of male respondents have supported and approved using and choosing family planning methods, but none of them practiced terminal methods. Generally, more male respondents disagreed than agreed that men should make decisions about selected family planning issues in the family. Decision-making dynamics around method choice followed a slightly different pattern. According to female participants, decisions regarding method choice were equally made by women or jointly, with male-dominated decisions falling last. There are many challenges to increase male involvement in family planning services. So far very few interventions addressing these challenges have been evaluated scientifically. Health education campaigns to improve beliefs and attitudes of men are absolutely needed. Additionally, improving accessibility, affordability, availability, accommodation and acceptability of family planning service venues will make them more attractive for male partners.

## Introduction

The World Health Organization (WHO) estimated in 2012 that 287,000 maternal deaths occurred in 2010; sub-Saharan Africa (56%) and Southern Asia (29%) accounted for the global burden of maternal deaths [1]. Countries still have to solve their rapid and uncontrolled increase in population [2]. It is well documented that men's general knowledge and attitude concern the ideal family size, gender preference of children, ideal spacing between child births, and contraceptive methods used greatly influence women's preferences and opinions [3–5].

The family planning method used can help ensure healthiest timing and spacing of pregnancy, hence, regulating fertility. As fertility falls, so do infant, child, and maternal mortality. Women spend decreasing proportions of their lifetimes giving birth and caring for young children [6]. Contraception plays a key role in decreasing maternal mortality. They provide significant protection for women by preventing unintended pregnancies, which often end in unsafe abortions [7]. However, fertility and family planning research programs have ignored men's roles in the past, focusing on women's behaviour, [5] and services are traditionally presented within the context of maternal and child health [8]. Since the 1994 International Conference on Population and Development (ICPD), and the 1995 UN World Conference on Women, interest in men's involvement in reproductive health has increased [5, 9]. There has also been a shift in objectives of male participation and concerns, from increasing contraceptive use and achieving demographic goals to achieving gender equality and fulfilling various reproductive responsibilities.

The large number of articles [10–12] and the growing number of conferences, research projects, and debates on this subject bear testimony to the importance of this issue, both from the programmatic point of view and as a process for bringing about a gender balance in men and women's reproductive rights and responsibilities. Men are also recognized to be responsible for the large proportion of ill reproductive health suffered by their female partners [2, 9]. Male involvement helps not only in accepting a contraceptive but also in its effective use and continuation [9]. Spousal communication on contraception and reproductive goals suggests that the couple has an egalitarian relationship [10]. Studies have shown that couples who discuss the number of children they desire or the use of family planning are more likely to use a contraceptive and achieve their reproductive goals than those who do not [6, 10]. This systematic review was aimed at determining the extent of male involvement in family planning decision making among sub-Saharan countries. The objectives were to assess men's knowledge, attitude, and practice of modern contraceptive methods; determine the level of spousal communication about family planning decision making; and investigate the correlates of men's opinion about their roles in family planning decision making.

## Methods

### Types of studies

Randomized controlled trials, controlled before and after, uncontrolled before and after, interrupted time series, cross-sectional studies, cohorts, and case control studies.

### Types of intervention

We will include men's attitude and practice about self/spousal use of family planning, spousal communication and men's opinions about family planning decision making.

### Types of outcome measures

The primary outcomes were male contribution in family planning decision making. Secondary outcomes include: men's knowledge of family planning methods, men's attitude and practice about self/spousal use of family planning, spousal communication and men's opinions about family planning decision making.

### Search methods for identification of studies

We searched the following electronic databases from January 1995 to December 2013: Medline, Embase (Excerpta Medica Database), CINAHL (Cumulative Index to Nursing and Allied Health Literature), LILAS (Latin American and Caribbean Literature on Health Sciences), International Bibliography of Social Sciences, Social Services Abstracts, and Sociological Abstracts. Along with MeSH terms and relevant keywords, we used the Cochrane Highly Sensitive Search Strategy for identifying reports of articles in PubMed. There were no restrictions to language or publication status.

### Effectiveness outcomes

Studies would be included if they provided data on the role of men in family planning decision making in sub-Saharan Africa.

### Data extraction and management

Two research team members (CDE and TNC) independently conducted data extraction from the final sample of articles by using a pre-established data extraction form. Disagreements were resolved by consensus or by arbitration of a third review author (VZM). Studies were reviewed for relevance based on types of participants (men), interventions (contribution in decision making in family planning), and outcome measures. We retrieved full text copies of the articles identified as potentially relevant by either one or both review authors. The flow of study selection is described in a Preferred Reporting Items for Systematic Reviews and Meta-Analyses (PRISMA) diagram [11]. Data are reported in a narrative manner.

### Assessment of quality in included studies

The included studies were not scored for quality.

## Current status of knowledge

### Results of the search

**Figure 1:**
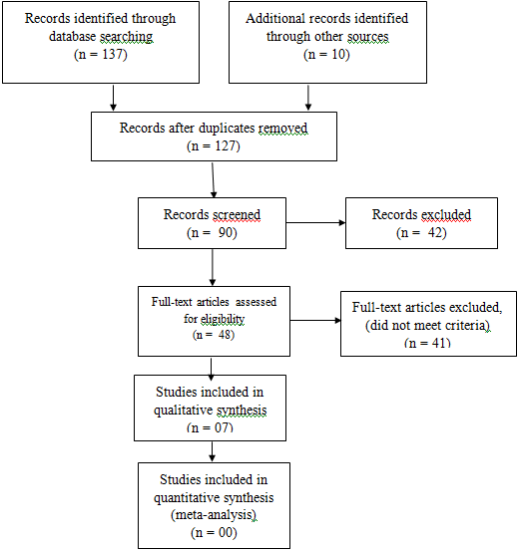
PRISMA flow diagram

Searches were conducted in January 2014 and identified 137 titles (Figure 1). Studies were reviewed for relevance based on: study design, types of participants, exposures and outcomes measures. Disagreements were resolved by discussion. Seven full-text articles were closely examined by authors, including a trial, identified by checking references.

### Awareness of family planning methods

Seven studies assess awareness of family planning methods in Nigeria [12–15] Ethiopia [16, 17] and Malawi [18]. In the Nigerian study, almost (99%) of men were aware of the existence of modern contraceptives, and most of them were aware of at least two modern methods. Awareness of the condom was highest (98%) [12, 14, 15]. The most popular source of information about family planning among them was the radio (93%) [12, 14]. In Ethiopia, the study on knowledge assessment revealed that most of the men respondents (75%) reported having knowledge on concepts and benefits of family planning methods. About 62% of male respondents listed two or above family planning methods, while only 14% of them able to list all the family planning methods used by men, 50% only one method mainly condom, while 30% did not know about the family planning methods to be used by men. About 40% men respondents reported 2 years and above the interval between two consecutive pregnancies, 31% between 1 and 2 years and 23% reported above 2 years [16]. The concept of family planning was well known to respondents: 760 (94%) women and 795 (98%) men responded ever having heard of it [17]. In Malawi study, all of the participants reported that they were not using contraception before the intervention. After the intervention, 78% per cent of the intervention arm and 59% of the comparison arm reported that they were using family-planning methods with their wives. Of those men in the intervention arm who reported family-planning uptake, 56% reported using condoms, and 41% and 14% reported using injectable and the birth-control pill, respectively [18].

### Men's attitude and practice about self/spousal use of family planning

Three studies assess men's attitude and practice about self-use of family planning in Nigeria [12, 15], Ethiopia [16, 17]. In Nigeria, 89% of men approved of their spouses using family planning while only 11% of them objected to it. However, almost two-thirds (65%) of the men disapproved of attending family planning clinics with their spouses, while only 26% of them had ever done so [12]. In Ethiopia, above 90% of male respondents have supported and approved using and choosing family planning methods, but none of them practiced terminal methods [16]. Long-term contraceptive methods were better known by women, and traditional methods as well as emergency contraception by men. In general only 4 out of 811 men ever used contraception, while 64% and 43% females ever used and were currently using contraception respectively [16]. Male respondents were asked specifically whether they would support their wives to use family planning. Of the 811 male respondents, 751 (93%) answered positively and 22 (3%) negatively [17].

### Spousal communication about family planning decision making

In Nigeria, consistently, less than a quarter of men individually initiated discussions on such issues as when to achieve pregnancy, when to avoid pregnancy, and the use of contraceptives in the year prior to the study [12, 14]. More than half (56%) of men, in Ethiopia reported no discussion with their wives on related issues of family planning use and believed that it is a natural process and need not to be discussed. However, 44% believed that discussion on these issues should be always initiated in the family. Similarly, 78% of them reported that decision were generally taken jointly with wife, while 21% felt that all decision related to family planning should be taken by wives alone. Another 12% felt that elder family members and relatives, external power should decide [16].

### Men′s opinions about their roles in family planning decision making

Generally, more male respondents disagreed than agreed that men should make decisions about selected family planning issues in the family. Forty-four per cent of men agreed that men should determine family size while 54% disagreed; 29% agreed that men should make the decision about when to adopt family planning while 69% disagreed; 9% of men agreed that men should decide which family planning method to adopt while 88% disagreed; 34% of men agreed that men should decide what to do about an unwanted pregnancy while 64% disagreed [12]. The next key landmark in the evolution of the Programme was its response to the International Conference on Population and Development (ICPD) held in Cairo, Egypt, in 1994. This conference highlighted the links between population, health and development, but also denounced the use of women as the sole subjects of population policies [19]. This review showed that different definitions of male involvement in family planning are used in different studies resulting in difficulties when comparing data between these studies. Determining a consensus definition of male involvement may be a necessary first step to measure efficacy and enhance comparability across programs [20]. In our review we identified two categories of factors associated with male involvement.

### Socio-demographic factors

Age and marital status: Most studies reported that older ages and cohabitation were associated with male involvement [12–15], Ethiopia [16, 17] and Malawi [18]. Health workers and the print media were the least mentioned planning methods at some time, less than 60 per cent of them were current users of any family planning method. Seventy-seven per cent of the men reported the condom as the family planning method ever used by their families [12]. A majority of women and men described the decision to use family planning method as being made by men, while only a minority of women said that male partners made the decision about what method they would use, a majority of men reported this pattern. In discussing method choice, men often described the reasons for choosing a particular method including cost, economic circumstances, and access [18].

### Socio-cultural factors Cultural

In several studies cultural standards were identified as barriers for male involvement [12, 16–18]. All respondents believed that reasons for involving men in family planning programmes include that men play dominant role in decision-making in the family. Other reasons stated by the majority of discussants are that men are family heads and exert a lot of influences on women's decision. Decision-making patterns described by the participants can take three main approaches: joint decisions, male-dominated decisions, and female-dominated decisions. As highlighted, male-dominated decisions were the norms across female and male participants, followed by joint decision making, and last, female-dominated decisions [18]. Looking separately at decisions to use family planning and decisions about contraceptives methods revealed more information about decision-making dynamics. Decisions to use family planning followed similar dynamics as described earlier. In one report, men who accompanied their wives to family planning services were perceived as being dominated by their wives [12]. Frequently, men perceive that family planning services are designed and reserved for women, thus are embarrassed to find themselves in such “female” places involvement [12, 16, 17]. In Nigeria, almost two-thirds (65%) of the men disapproved of attending family planning clinics with their spouses, while only 26 per cent of them had ever done so [12].

### Communication

Poor communication between men and their female partners was associated with poor male involvement. Furthermore, 35% of men reported never discussing family planning issues with their spouses in the year preceding the survey. However, 49% of men reported discussing family planning at least once or twice during the same period [12, 14]. In Malawi, many interview participants said that overall communication with their wives or girlfriends was enhanced by their increased comfort with discussing family planning, which some attributed directly to their interactions with the male motivator [18]. In Nigeria, consistently, less than a quarter of men individually initiated discussions on such issues as when to achieve pregnancy, when to avoid pregnancy, and the use of contraceptives in the year prior to the study [12].

### Limitations

A formal meta-analysis was not feasible for this topic. Most of the studies were descriptive in nature; and none of the review studies was found to be methodologically stronger according to our quality ratings. While a meta-analysis would be meaningless in the face of such disparate studies, it is nonetheless revealing that key themes were consistent across continents, populations, and study designs, suggesting their robustness. Some of the data presented in this study where absolute numbers, in systematic reviews such vote counting is known to frequently bias the interpretation of findings, since this ignores the effect size and sample size of the studies. However, such counting is mainly used to categorize the study characteristics not to report any study outcomes as such.

## Conclusion

There are many challenges to increase male involvement in family planning services. So far very few interventions addressing these challenges have been evaluated scientifically. Health education campaigns to improve beliefs and attitudes of men are absolutely needed. Additionally, improving accessibility, affordability, availability, accommodation and acceptability of family planning service venues will make them more attractive for male partners.
